# Femtosecond X-ray diffraction from two-dimensional protein crystals

**DOI:** 10.1107/S2052252514001444

**Published:** 2014-02-28

**Authors:** Matthias Frank, David B. Carlson, Mark S. Hunter, Garth J. Williams, Marc Messerschmidt, Nadia A. Zatsepin, Anton Barty, W. Henry Benner, Kaiqin Chu, Alexander T. Graf, Stefan P. Hau-Riege, Richard A. Kirian, Celestino Padeste, Tommaso Pardini, Bill Pedrini, Brent Segelke, M. Marvin Seibert, John C. H. Spence, Ching-Ju Tsai, Stephen M. Lane, Xiao-Dan Li, Gebhard Schertler, Sebastien Boutet, Matthew Coleman, James E. Evans

**Affiliations:** a Lawrence Livermore National Laboratory, 7000 East Avenue, Livermore, CA 94550, USA; b Department of Molecular and Cellular Biology, University of California, Davis, 1 Shields Avenue, Davis, CA 95616, USA; c Linac Coherent Light Source, 2575 Sand Hill Road, Menlo Park, CA 94025, USA; d Arizona State University, 300 East University Drive, Tempe, AZ 85287, USA; e Center for Free-Electron Laser Science, University of Hamburg, Luruper Chaussee 149, Hamburg 22761, Germany; f Center for Biophotonics, 2700 Stockton Boulevard, Sacramento, CA 95817, USA; g Paul Scherrer Institute, 5232 Villigen PSI, Switzerland; h Environmental Molecular Sciences Laboratory, Pacific Northwest National Laboratory, 3335 Innovation Boulevard, Richland, WA 99354, USA

**Keywords:** two-dimensional protein crystal, femtosecond crystallography, single layer X-ray diffraction, membrane protein

## Abstract

X-ray diffraction patterns from two-dimensional (2-D) protein crystals obtained using femtosecond X-ray pulses from an X-ray free-electron laser (XFEL) are presented. To date, it has not been possible to acquire transmission X-ray diffraction patterns from individual 2-D protein crystals due to radiation damage. However, the intense and ultrafast pulses generated by an XFEL permit a new method of collecting diffraction data before the sample is destroyed. Utilizing a diffract-before-destroy approach at the Linac Coherent Light Source, Bragg diffraction was acquired to better than 8.5 Å resolution for two different 2-D protein crystal samples each less than 10 nm thick and maintained at room temperature. These proof-of-principle results show promise for structural analysis of both soluble and membrane proteins arranged as 2-D crystals without requiring cryogenic conditions or the formation of three-dimensional crystals.

## Abstract 2

Bragg diffraction achieved from two-dimensional protein crystals using femtosecond X-ray laser snapshots is presented.

## 1. Introduction   

X-ray crystallography has been the leading method for atomic resolution structure determination of biological macromolecules since the 1950s (RCSB, 2013 ▶), yet this technique is typically limited to macroscopic three-dimensional (3-D) protein crystals larger than 10 µm per side (Holton & Frankel, 2010 ▶) when using synchrotron light sources. However, some proteins, including membrane proteins, are observed to form two-dimensional (2-D) crystals, a sample geometry that to date has not been suitable for forward-scattering X-ray analysis due to limitations of radiation damage. Grazing-incidence X-ray diffraction (GIXD) has permitted the collection of X-ray powder diffraction patterns from 2-D protein crystals at the air–water interface, but this technique uses reflected, not transmitted, X-rays and the typical beam footprint (between 5 and 100 mm^2^) is much larger than the average 2-D crystal grain size (∼75 µm^2^ for streptavidin) resulting in the simultaneous probing of multiple, not individual, 2-D crystals (Lenne *et al.*, 2000 ▶; Verclas *et al.*, 1999 ▶). While transmission electron microscopy has yielded protein structures from 2-D crystals of both soluble proteins (Nogales *et al.*, 1998 ▶) and membrane proteins (Gonen *et al.*, 2005 ▶; Henderson *et al.*, 1990 ▶), fewer than 30 unique structures have been solved to better than 4 Å with this technique. For each of these above-mentioned methods, achieving high-resolution structures from 2-D crystals can be significantly hindered by radiation damage.

The recent commissioning of X-ray free-electron lasers (XFELs), such as the Linac Coherent Light Source (LCLS), has enabled successful structure determination by serial femtosecond crystallography from protein nanocrystals as small as 200 nm (Boutet *et al.*, 2012 ▶; Chapman *et al.*, 2011 ▶). The extremely short femtosecond-duration pulses delivered by XFELs have a peak brightness many orders of magnitude greater than synchrotron sources permitting the collection of X-ray diffraction (according to simulations) from even smaller samples including 2-D crystals (Kewish *et al.*, 2010 ▶) and single particles (Neutze *et al.*, 2000 ▶) at doses significantly exceeding the normal tolerable room-temperature radiation dose (Redecke *et al.*, 2013 ▶). Transmission X-ray diffraction from monolayer 2-D protein crystals using an XFEL could provide a new approach for structure determination of proteins that fail to readily form macroscopic 3-D crystals. In particular, this approach may benefit structure determination of membrane proteins that can be grown into 2-D crystals embedded within a lipid bilayer which mimics their native environment and avoids additives used for *in surfo* 3-D crystallization that may perturb the native protein conformation or functionality (Srivastava *et al.*, 2012 ▶). Additionally, 2-D crystals possess a compact support along the beam direction which presents entirely new possibilities for solving the phase problem iteratively in 3-D (Spence *et al.*, 2003 ▶).

## 2. Methods   

### 2.1. Silicon wafers   

200 µm-thick silicon dice with thin silicon nitride membranes were purchased from Silson, Inc. Two types of dice were used for this work. The die used for the streptavidin sample measured 25 mm × 25 mm with a 15 × 29 array of 200 µm × 750 µm membranes (50 nm thick). The die used for the bacteriorhodopsin sample measured 11 mm × 25 mm with a 12 × 29 array of 200 µm × 200 µm membranes (30 nm thick).

### 2.2. Streptavidin   

Streptavidin crystals were grown at the air–water interface using the lipid-monolayer approach as previously described (Darst *et al.*, 1991 ▶). Briefly, a lipid solution dissolved in chloroform and composed of 80% DOPC (Avanti Polar Lipids) and 20% biotinyl-cap DOPC (Avanti Polar Lipids) was deposited at the air–water interface of a Teflon plate (Lévy *et al.*, 1999 ▶). After stabilization of the continuous lipid monolayer, streptavidin (Sigma Aldrich) was injected into the buffered sub-phase at a final concentration of 0.1 mg ml^−1^ (buffer: 50 m*M* Tris, pH 7.0, 150 m*M* NaCl). After 30 min, the crystals were harvested by adhering them to the surface of a silicon nitride membrane coated with an octadecyltrichlorosilane (OTS) monolayer (Sung *et al.*, 1999 ▶). For structural preservation, the samples were sugar embedded using a modified carbon sandwich technique (Gyobu *et al.*, 2004 ▶) wherein the harvested crystals were incubated with a 2% sucrose solution, covered with a 10 nm-thick continuous carbon film and excess solution removed by holding the die vertical and wicking from the bottom.

### 2.3. Bacteriorhodopsin   

Purple membranes (PM) were isolated from *H. salinarum* strain S9 using previously described procedures (Sonar *et al.*, 1994 ▶) and diluted to 12, 6, 3 and 1 mg ml^−1^ prior to use. Purple membrane was also treated with varying concentrations of 0–10% of detergent in PBS to break up the PM into smaller soluble bacteriorhodopsin patches (Nollert *et al.*, 2001 ▶). A total of 0.5 µl of each concentration was sequentially dropped onto the silicon die and allowed to air dry.

### 2.4. Femtosecond X-ray diffraction   

Femtosecond X-ray diffraction was performed using the Coherent X-ray Imaging (CXI) instrument (Boutet & Williams, 2010 ▶) of the Linac Coherent Light Source. Once the dice with 2-D crystals were mounted on the CXI sample stage the samples were probed using X-ray pulses consisting of nominally 1.9 × 10^12^ photons within a beam less than 300 nm in diameter that was produced using Kirkpatrick–Baez focusing mirrors. While the initial X-ray pulse length was based on the measured electron bunch length and estimated to be 50–60 fs, newer more direct measurements of the X-ray pulse length indicate the actual X-ray pulse length to be ∼30 fs. An X-ray wavelength of 1.462 Å corresponding to a photon beam energy of 8448 ± 50 eV was used for all experiments and the detector distance was set at 560 mm (for streptavidin) and 340 mm (for bacterio­rhodopsin). Diffraction patterns were recorded using the Cornell-SLAC Pixel Array Detector (CSPAD) and sorted using *Cheetah* (Barty, 2013 ▶), *Matlab* and *CrystFEL* (White *et al.*, 2012 ▶) software suites.

### 2.5. Diffraction pattern analysis   

Owing to the use of a non-tilting sample stage, diffraction patterns were assumed to be untilted and normal to the X-ray pulse. *Matlab*, *CCP4* and *UCSF Chimera* were used for all analysis. Initially, the diffraction patterns were overlaid with expected lattice positions calculated from assumed unit-cell parameters of *a* = *b* = 82 Å and α = β = γ = 90° with *C*_222_ symmetry for streptavidin and *a* = *b* = 63 Å and α = β = 90°, γ = 120° with *P*_3_ symmetry for bacteriorhodopsin. These unit-cell parameters were derived from both transmission electron microscope data of similarly prepared samples and from previous publications (Darst *et al.*, 1991 ▶, Henderson *et al.*, 1990 ▶, Lenne *et al.*, 2000 ▶, Verclas *et al.*, 1999 ▶). For both samples the expected lattice unit-cell parameters for an untilted crystal closely matched the observed reflections and were used as a local marker for subsequent peak searches. It should be noted that in the case of streptavidin, the *C*_222_ symmetry results in systematic absences for reflections where the indices *h* + *k* = 2*n* + 1, so the innermost spots within the streptavidin lattice represent the (0, 2), (1, 1) and (2, 0) reflections not (0, 1), (1, 1) and (1, 0). Localized peak searches were then performed using the expected lattice positions as the central starting locations and only peak values equal to or greater than 7 ADU (analog-to-digital units) were considered valid peaks since a single 8.4 keV photon yields a pixel value of approximately 8 ADU on the CSPAD. The integrated intensity for each identified peak was calculated by using a 3 × 3 pixel search to identify all connected pixels with values greater than 7 and summing them together with the central peak. Only integrated intensities greater than 15 were included in subsequent analysis since this provided a signal-to-background ratio of at least 5. To estimate the electron density projection map from the observed reflections of each single-shot pattern, a generalized molecular replacement scheme was used, wherein the CCP4 program *SFALL* was first used to generate a list for all calculated reflections (*H*, *K*, *L*, *F*_*c*_ and phase) to 8 Å resolution from the known structures of bacteriorhodopsin (PDB: 2ntu) and streptavidin (PDB: 3rdx). A final experimental reflection list was created by combining the *H*, *K*, *L* and corresponding integrated intensity for each experimentally observed spot with the calculated phase (PHIC) values generated by *SFALL* for the corresponding calculated reflections. The CCP4 program *F2MTZ* then converted the experimental reflection lists from *HKL* to MTZ format and the resulting spot lists were input into the CCP4 program *FFT* to yield a 2-D electron density projection map of a 2 × 2 unit cell. *UCSF Chimera* was used to visualize the 2 × 2 unit-cell ribbon diagrams of the known structures for comparison.

## 3. Results   

**Figure 1:**
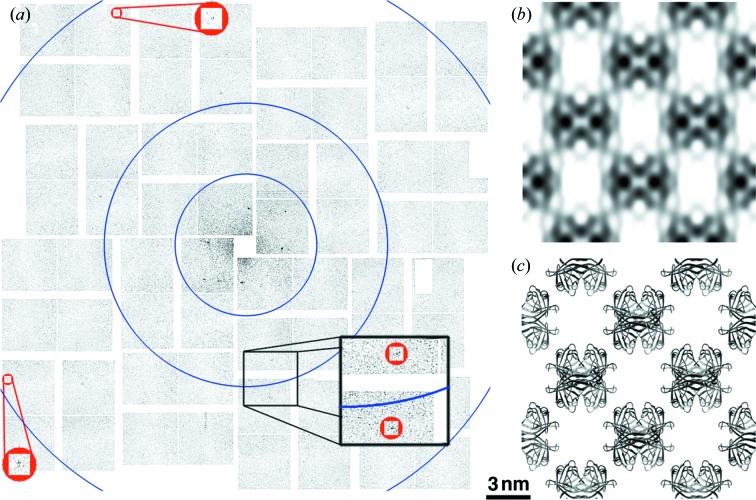
Bragg diffraction at sub-nanometer resolution from soluble protein 2-D crystals. (*a*) Background-subtracted diffraction pattern for 2-D crystals of streptavidin. Blue circles signify resolution rings at 30.0, 15.0 and 7.5 Å (inner to outer). The zoomed-in red circles indicate Bragg spots with highest resolution at 8.0 Å, (*h*, *k*) = (−10, 2) and (2, 10), while the black box zoom highlights two lattice spots at intermediate resolution. The diffraction patterns were acquired with a sample-to-detector distance of 560 mm and a photon energy of 8448 eV. Owing to *C*_222_ symmetry (*h* + *k* = 2*n*), the innermost reflections are (0, 2), (1, 1) and (2, 0). (*b*) 2-D electron density projection map (2 × 2 unit cells) from coupling the observed integrated peak intensities with the corresponding calculated phases from the known crystal structure. (*c*) Ribbon diagram of a 2 × 2 unit cell of streptavidin created using the known crystal structure, symmetry and unit cell for comparison with (*b*). The scale bar is equivalent for panels (*b*) and (*c*).

For our experiments, 2-D crystals of streptavidin (soluble protein) and bacteriorhodopsin (membrane protein) were prepared as fixed targets and adhered to thin-film silicon nitride windows 30–50 nm-thick on a silicon wafer support. This fixed target design acts as a solid support to promote crystal flatness while contributing only a small X-ray scatter background that was mostly confined to low angles. Furthermore, the solid support minimizes consumed sample volume by requiring only a few micrograms of dispersed 2-D crystals to prepare a fixed target for analysis. Diffraction patterns were collected by aligning the fixed target windows into the 0.1 µm focus beam path of the CXI instrument at LCLS (Boutet & Williams, 2010 ▶) in vacuum; each window was exposed to a single ∼30 fs pulse of 8448 ± 50 eV X-rays with a power density exceeding 7 × 10^19^ W cm^−2^ and diffraction measured using the CSPAD detector. Figs. 1(*a*) ▶ and 2(*a*) ▶ show single-shot femtosecond diffraction patterns measured from individual 2-D crystals of streptavidin and bacteriorhodopsin at room temperature. Each pattern demonstrates Bragg diffraction to better than 8.5 Å surpassing the best resolution reported in previous GIXD powder diffraction patterns from similar 2-D crystal preparations of streptavidin (Lenne *et al.*, 2000 ▶) and bacteriorhodopsin (Verclas *et al.*, 1999 ▶) at 13.0 and 8.8 Å, respectively. Diffraction from individual monolayer crystal domains rather than an ensemble of crystals was possible due to the use of an X-ray beam 500 million times smaller than that required for GIXD (Verclas *et al.*, 1999 ▶).

**Figure 2:**
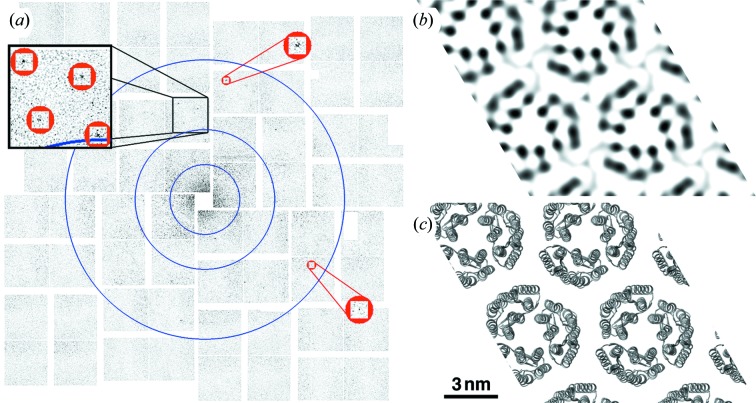
Bragg diffraction at sub-nanometer resolution from membrane protein 2-D crystals. (*a*) Background-subtracted diffraction patterns for 2-D crystals of bacteriorhodopsin. Blue circles signify resolution rings at 30.0, 15.0 and 7.5 Å (inner to outer). The zoomed-in red circle highlights the peaks with highest resolution at 8.5 and 8.7 Å, (*h*, *k*) = (2, 5) and (3, 4), respectively. The diffraction patterns were acquired with a sample-to-detector distance of 340 mm and a photon energy of 8448 eV. (*b*) Experimental 2-D electron density projection map (2 × 2 unit cells) from coupling the observed integrated peak intensities with the corresponding calculated phases from the known crystal structure of bacteriorhodopsin. (*c*) Ribbon diagram (2 × 2 unit cells) of bacteriorhodopsin created using the known crystal structure, symmetry and unit cell for comparison with (*b*). The scale bar is equivalent for panels (*b*) and (*c*).

**Figure 3:**
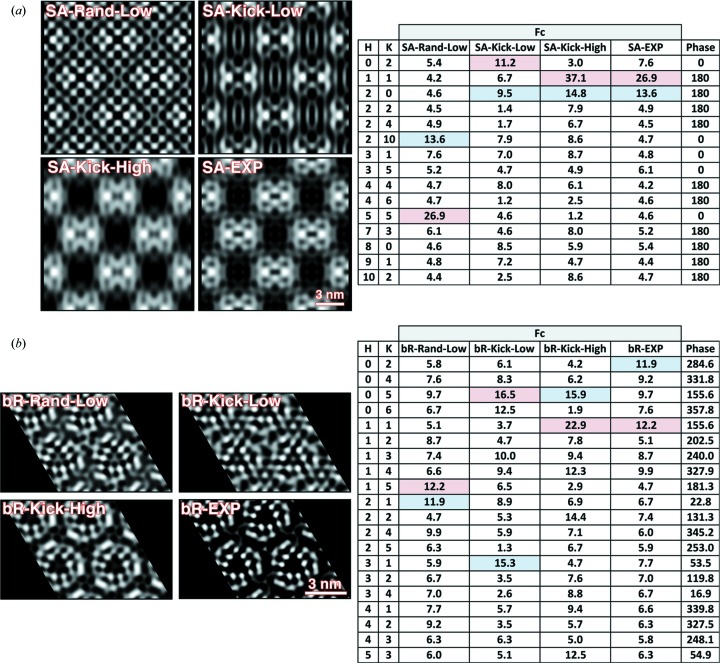
Evaluating the quality of experimental projection maps by randomly varying the observed peak amplitudes. The *F*_*c*_ columns of the *HKL* files used to generate the projection maps in Figs. 1(*b*) ▶ and 2(*b*) ▶ were extracted using *Matlab* and either randomly rearranged (relative to their associated reflections and phases) or randomly kicked (modulated) by factors ranging from 50% to 300%. Ten iterations of each randomization method were performed and three of the 20 resulting maps are shown for streptavidin (*a*) and bacteriorhodopsin (*b*). File names are displayed for each iteration and ‘rand’ represents the random rearrangement tests while ‘kick’ represents the random modulation tests. The projection maps from the experimentally measured amplitudes ‘exp’ are shown (bottom right) for comparison. For both streptavidin and bacteriorhodopsin, the experimentally measured projection map had the highest match (cross-correlation) to the known structure. In some of the randomized maps the correlation coefficient approaches the level seen for the experimentally measured maps (‘high’) but the majority of maps show significant differences in the projected density and had low correlation (‘low’). Tables comparing the *HKL* file details corresponding to each map are shown on the right. For quick visualization of the amplitude hierarchy, the highest and second highest *F*_c_ value for each map is highlighted in the table in light red and light blue, respectively. While randomized amplitude tests that gave rise to high map correlations maintained the overall hierarchy of the experimentally measured amplitudes for low-order reflections (which strongly influence the overall density distribution), disrupting the amplitude hierarchy as seen in the low correlation maps (either through amplitude rearrangement or modulation) adversely affects the resulting projection maps. This suggests that even though a single-shot measurement with LCLS will yield structure factors with large error bars due to the fluctuating source parameters that ultimately need to be averaged over multiple crystals to converge to reliable values, the presented results clearly yield better structures than randomized Bragg intensities and therefore the intensities measured are not random.

Diffraction patterns from individual 2-D crystals were indexed and the integrated peak intensities used for single-pattern molecular replacement to generate non-tilted 2-D electron density projection maps [Figs. 1(*b*) ▶ and 2(*b*) ▶]. Both maps show the appropriate unit cell and symmetry as compared with known structures [Figs. 1(*c*) ▶ and 2(*c*) ▶] but, since each individual diffraction pattern does not include every reflection, the resulting projection maps show some added density on two sides of the streptavidin molecules (Fig. 1*b* ▶) and symmetry amplified density at the threefold rotational center of the bacteriorhodopsin unit cell (Fig. 2*b* ▶). Owing to the minor variations relative to model structures, we examined the potential effects of phase bias on our projection electron density maps by conducting a series of sensitivity tests wherein the experimental amplitudes were randomized relative to model phases or where the amplitudes were randomly modulated (Fig. 3 ▶). However, these tests verified that the projection maps generated using the experimentally determined amplitudes exhibited the highest correlation with model structures indicating that the observed Bragg reflections from single patterns contain structural information.

## 4. Discussion   

Transmission X-ray diffraction patterns from individual sugar-embedded 2-D protein crystals highlight the potential for XFELs to enable the study of 2-D crystals of biological macromolecules including undamaged membrane proteins and soluble proteins at room temperature. Some of the collected patterns from bacteriorhodopsin samples exhibited multiple lattices that were a result of diffraction from multilayer crystal stacking. The stacking could have been caused by inherent sample interactions or due to the concentration of 2-D crystals deposited on the substrate. The extent to which multiple lattice or powder diffraction XFEL data can be reliably used for structure determination from 2-D crystals is currently unclear. However, it is interesting to note that all the diffraction patterns acquired during this initial experiment were limited to ∼8 Å regardless of whether the patterns showed single or multiple lattices.

We believe that the 8 Å diffraction achieved here does not indicate the maximum attainable resolution for this method but rather signifies that additional improvements in sample preparation, experimental conditions and data analysis continue to be needed. Future optimization of crystal patch size and quality, single layer crystal coverage on support film, method of sample preservation (sugar embedding of varied composition and concentration *versus* fully hydrated or cryogenic), and the flatness/rigidity of the support film may each permit higher-resolution XFEL data collection from 2-D crystals. Additionally, while the ∼30 fs pulses used here demonstrate the ability to outrun damage, at least to ∼8 Å, it is possible that even shorter pulses may be required to prevent high-resolution component degradation either from direct beam interactions with the sample or potentially from photoelectrons generated by the support film. At the same time, the pulses themselves may be fast enough to maintain the high-resolution components but might not have enough photon density for sufficient scattering at high angles so a smaller beam size or increased brightness could also yield better diffraction. Finally, new algorithms for data analysis could evaluate whether merging of large datasets might reveal higher-resolution information that is already present in the data but currently appears buried within the noise for individual patterns.

While any one, or a combination, of the above experimental considerations may improve the attainable resolution, a tilting stage will be needed to collect data for 3-D reconstructions from 2-D crystals. Our presented results only include diffraction patterns acquired at zero-degree tilt (normal incidence to 2-D crystal plane) due to physical limitations of the available fixed target sample stage at the time of these experiments. As in conventional X-ray approaches for 3-D crystals, the phase problem remains one of the largest challenges for XFEL imaging of 2-D crystals. The lack of tilted data restricted us to using a single-pattern molecular replacement method to simulate the electron density maps; however, the compact nature of 2-D crystals may permit new approaches for direct phasing (Spence *et al.*, 2003 ▶) when tilted data are available. Furthermore, hybrid approaches may also provide an answer to the phase problem. Electron crystallography of 2-D crystals already combines the diffraction pattern peak intensities with phase information derived from images of equivalent crystals (Gonen *et al.*, 2005 ▶) and a similar approach may be possible to enhance XFEL data with phase information from electron microscopy. The incorporation of fixed target sample stages with tilting capability and hybrid or novel approaches for phasing should therefore dramatically expand the utility of imaging 2-D crystals with an XFEL for structural biology.

Overall, the proof-of-principle results presented here establish diffraction-before-destruction as a new strategy for structure determination from individual 2-D protein crystals, including membrane proteins that currently represent less than 2% of solved atomic resolution protein structures (White, 2013 ▶) despite constituting over 25% of all proteins in nature (Krogh *et al.*, 2001 ▶). Although the single-shot diffraction patterns presented here were from static samples, they demonstrate the ability to perform studies at room temperature, which is a critical step towards future time-resolved pump–probe experiments of 2-D crystals since physiologically relevant dynamics generally occur at or above room temperature. Bypassing the need to freeze 2-D crystals, as currently used for high-resolution imaging with cryo-electron microscopy (Chou *et al.*, 2007 ▶), may allow future XFEL diffraction studies of 2-D crystals to capture fast conformational changes of membrane proteins in a near-native environment and in real time.
